# Relation of Food to the Developing Teeth*Read before the California State Dental Association, June 20 1921. Printed in *Pacific Dental Gazette*, November, 1921.

**Published:** 1921-12

**Authors:** M. Evangeline Jordon


### RELATION OF FOOD TO THE DEVELOPING TEETH*

BY M. EVANGELINE JORDON, D.D.S.

Whatever may be said for or against the vitamine theory, as shown in laboratory experiments by Dr. Howe and others, it more clearly explains the conditions found in the mouths of children than any other theory so far advanced.

From the first of my work in college I had more than the average number of children patients, because even that early in my professional life the men in my class were glad to be rid of them so that they could settle down to the serious work of dentistry,—the making of bridges and plates. Thus having a number to observe, I began to see the difference in children living under different environments. Desiring to get more experience in extraction, which at that time was not in the hands of specialists, I went once a week to the Maria Kip Orphanage, and seeing so many teeth lost *"Made me to think,"* as the French say. To me it seemed a great economic waste to find the permanent teeth of these young children breaking down, thereby leaving the masticatory apparatus crippled for life. During my college course I was forced to teach again, in order to obtain funds to complete my dental education. Remembering my experiences at the Maria Kip, I began studying the mouths of the children, what they ate, and the effect of good or bad teeth upon their general health. I found that children who were on a properly balanced diet and who were allowed *no candy* developed sound, perfect and well-shaped arches, while children who were always munching candy had weak, undeveloped jaws, carious teeth, and as a rule frail bodies. These children were ir-

* Read before the California State Dental Association, June 20, 1921. Printed in *Pacific Dental Gazette,* November, 1921.

ritable in temper and backward in classes. Later, when I began limiting my work to children, I found that cement fillings would not last in certain mouths and that these children were sugar and starch eaters and the length of the filling was in direct ratio to the preponderence of carbohydrates in the diet. It is not only the evil effect of candy eaten by the child that must be considered, but any child who makes a practice of eating candy refuses the plain foods, which are *so* necessary in giving to the system the lime salts required by the growing body and developing teeth—ten times as much as will be necessary later in life. Even a child who eats normally, but drinks distilled water may show a deficiency of lime salts in tooth development.

DIET DURING PREGNANCY

From the first I realized the value of mastication in the development of facial muscles. I prescribed dark breads and vegetables and then found that caries could be controlled and often entirely prevented. In a large number of cases, where the utmost care had been given the small child's mouth and particular attention paid to its diet, I found defective fissures in the erupting first permanent molars. This condition proved conclusively to me that the diet of the pregnant mother was at fault.

Lately, I have been trying to get sufficient data, having in mind the report made by Hanan nearly thirty years ago that the bones of pregnant women who had enjoyed apparent health were the site of lesions resembling osteomalasia. ''Recently Hart, Stembaugh and Humphrey have confirmed these observations (respecting deficiency of lime in cattle) by careful experiments, which showed how the mere addition of calcium to the fodder of cows prevented the birth of premature, weak or dead calves. Indeed, the investigation of Forbes showing that cows producing large amounts of milk and fed *common winter* rations undergo constant losses of calcium, magnesium and phosphorus from their skeletons, suggests that large numbers of milk cows are suffering from a deficiency disease."

It is very apparent that many pregnant women also suffer from a deficiency disease, which is indicated by the lack of health of the offspring. This happens when children are born in rapid succession whereby the mother has not had sufficient time to recover from her previous condition. Such children often show scurvy and rickets, also develop weak, flabby gums, and poor teeth which are irregular and show marked hypoplasia. Hypoplasia of the deciduous teeth is not common, but several cases have been brought to me. In every case I learned that the mother, during pregnancy, had suffered a severe illness.

Even when breast-fed, the mother's milk may not have been sufficiently rich in lime salts and vitamines to develop a strong, healthy child. Often the child is put on the bottle and given a prepared food, overly sweet, or milk so sterilized that the vitamines are destroyed, and scurvy or rickets is the result. Hess says that rickets is the most common nutritional disease occurring among children of the temperate zone. Fully three-fourths of the infants of large cities like New York show rachitic signs to some degree and it is a most overlooked disease.

The first permanent molar bears the mark of hypoplasia more frequently than any other tooth in the dentures, and this may, in part, account for the large number of these teeth that are lost early in life. Only the most painstaking work and constant supervision will protect such teeth which are highly sensitive and difficult to fill. Let me pause here to say that copper amalgam is most valuable in filling each tiny break in the enamel so that the tooth may again play its part in mastication until gold may be used to restore the lost contours.

The injury to the permanent molar may have occurred before birth or during the first year.

### BOTTLE-FEEDING OF INFANTS

When I find teeth showing hypoplasia, I nearly always am told that the child was bottle-fed. These malformed teeth are only the extreme cases. Many children show scurvy, which is to be recognized by the diagnostic signs of swollen, bleeding gums overlapping the erupting teeth, often a dark purple in color. Such children are nervous and fretful because of the pain they suffer in their joints. Put upon raw milk and orange juice they make a rapid recovery, and if foods rich in vitamines are continued, there will be no recurrence of the scurvy.

The pasteurizing of milk is receiving considerable attention, as when it is carelessly done it may be one of the factors in deficiency feeding that will result in scurvy. Hess says that milk heated to 145 degrees F. for thirty minutes has lost more of its anti-scurvy potency than milk that has been raised to 212 degrees F. for a few minutes. The result confirms clinical experience that scurvy occurs more frequently on a diet of pasteurized milk than one of boiled milk.

### DIET OF THE RUN-ABOUT

The bottle-fed infant already has an artificial appetite and it generally refuses to eat the hard foods necessary for the development of jaws and teeth. The average mother feeds it on starches such as rolled oats and cream of wheat, which she sweetens; fermentation in the alimentary canal is the usual result. The saliva is no protection to the tissues, which lack resistance. Caries of the teeth develop in the second or third year. Early infection of the tissues of the mouth may be wide-spreading, affecting the tonsil and glands and even the ears and eyes. The gums become soft and spongy and unless conditions are corrected result in the early symptoms of the condition termed pyorrhea. Such children suffer from anemia. I believe that the large percentage of school children suffering from under-nourishment are the victims of unwise diets more often than from lack of food. The parents have no education upon food values, thereby permitting each child to select its own diet to an alarming degree.

Did it ever occur to you how absurd it is to allow a small child whose judgment is warped by eating free sugar, now classed as a form of alcohol, to select its own diet? What raiser of stock would leave the oat bin open and urge his live stock to go in and glut themselves. Would he be astonished at the langour resulting and complain that there was a loss of appetite as hundreds of mothers do over their children? I am not laying all the blame on the mother, as I have repeatedly known fathers so blind as to bring home candy and also allow young children to eat anything upon the table. I also find such fathers leaving the nursing to mothers when outraged nature takes her revenge.

Dr. Bell, in his paper entitled, "Teeth, Tonsils and Toxemia in Relation to Diseases of the Eye," says: "Education along dental lines and in dental hygiene is just as necessary as education of the mind. Children who know nothing about the baleful effects of sugar and candies are constantly coming to my clinic."

In my office, when I question the parents as to the child's diet, in the majority of cases I get the following answers. Does the child eat vegetables? Yes, he nearly lives on potatoes, but will sometimes eat peas." Does he eat fruit? "Yes, he has several bananas a day." What does he have for breakfast? "Well, he won't eat much. He has a cup of chocolate very sweet and some white crackers or toast." You can see that the selective diet is nearly all starch and sugar. Bananas are the least digestible of fruits. Such children can not digest milk because of the fermentation in the alimentary canal. The parent is generally glad to be enlightened upon the bad effects of sugars and starches, and where a correct diet of milk, eggs and dark breads, fruit and vegetables is followed a remarkable change in health of the child results.

DIET IN RELATION TO THE TEETH

It is appalling to think that ninety per cent of the children of our country are suffering from caries of the teeth, which is a *preventable* disease. My twenty years of clinical experience leads me to believe it is largely a question of food. I have watched children upon a simple, wholesome diet, with little dental care, developing perfect teeth, and others upon the food of the average well-to-do American family whose teeth, despite every safeguard, will break down. In talking to a parent I first name all of the foods which must be limited and at the top of the list stands sugars and starches. I explain why no child can expect to have good teeth if he eats an excess of carbohydrates and then carefully go over the list.

No soft food, no white bread or crackers, no white cereals, a small amount of potatoes, no hot drinks, no candy or chocolate, no gum chewing, no waffles or hot cakes.

At once I am asked, "Well, what can we have," as I have forbidden about all that a child generally eats, and this list follows:

All kinds of dark bread, preferably bran; fresh fruit and vegetables; eggs and milk; some meat and fish; nuts, raisins, figs and dates.

Every meal should contain some roughage, raw vegetables or fruits and coarse bread. It is important that a pint or more of milk should be taken by a child daily. When one whose appetite has been vitiated by sweets has no desire for milk, it may be given over shredded wheat biscuits or in custard or milk soups served with dry toast. Very few articles of food should be served at one meal but a constant change should be made as a spur to the appetite so that plain food will be attractive. There is room for much education of the laity upon wholesome diet for children and no dietician has so far given the aid necessary, but if the dentists make known the urgent necessity of a diet rich in lime salts to protect coming generations so that nine out of every ten school children shall not suffer with their teeth some one will arise to fill this need.

In talking to the parent I never accept the excuse that a child inherits soft teeth, but explain that the child inherits or more correctly imitates *habits* that make soft teeth and that immunity to caries can only be obtained through food thoroughly masticated and with this aid the entire system will functionate properly. There will be little danger of undeveloped jaws or mal-placed teeth. The important point to be made is that every mouthful should be thoroughly masticated. To insure this a child should not have water at the table, but should be given a glass of milk to drink slowly at the end of the meal. It would be cruel to expect a child to masticate if the teeth are not in good repair. To put them in condition will require a much larger force of operators in the dental clinics or else the dental profession must prepare themselves to do the work. The time to cultivate a taste for vegetables is when the diet of the child is being changed from milk. If the child has been bottle fed, in most cases it already has an appetite for sweets, difficult to overcome.

Many physicians have a habit of giving children easily-digested foods, in a soft form, after the children have teeth with which to masticate, as well as a digestive organ which to be of any value must be exercised.

The result is a breakfast of cream of wheat loaded with sugar, a cup of chocolate, also very sweet, and a soft piece of toast of which the crusts are left out. Luncheon, a chop and mashed potatoes, a sweet dessert and several glasses of ice water. Dinner, more mashed potatoes, and chocolate to drink with cake or a sweet rice pudding. Add to this candy between meals and you have a truly sweet picture of the lack of good judgment in feeding the young of our free nation. What percentage of growth can you expect upon a diet so deficient in vitamines?

When brought for dental work such children present a picture that Luther Burbank has named "starch babies,"

and they usually have a useless dental equipment in the third or fourth year. When such children are put upon a wholesome diet, dry enough to require thorough mastication an immediate improvement is seen. Why not substitute a breakfast of fruit juices, bacon and eggs and hard toast; or shredded wheat biscuit and milk, stewed apples or prunes and hard toast. For luncheon, a chop or steak, peas or spinach or asparagus and a baked potato, alternating with rice or hominy gritz used as a vegetable and a fruit or vegetable salad. And at night a simple meal of vegetable soup with a crusty roll or the more old-fashioned dark bread and milk with fruit, and oatmeal or graham cookies. This will enable the salivary glands to supply an alkaline saliva.

### PROPHYLAXIS

As soon as I began to limit my work to children I found that supervision of my patients at frequent intervals was absolutely necessary and I began prophylaxis at regular periods. When I began, the idea of the oral hygienist had not been conceived and I met with some criticism especially by the type of dentist who told his patients he was too busy to clean their teeth and for them to go home and clean them themselves. Notwithstanding this the parents soon saw that their children were benefitting from this new form of dentistry and many availed themselves of it with the result that years later the children have gone back to their family dentist with almost perfect teeth in good occlusion. My plan is to give monthly prophylaxis extending or shortening the time as the mouth responds to treatment. The method is simple and requires only ten to twenty minutes. Paint the teeth and any portion of the surrounding soft tissues that appear abnormal, with iodin, then polish the teeth with a rubber cup and pumice. Follow with a wooden point in a hand porte polisher and dental floss. Finish by a thorough polishing of surfaces with a cotton roll dipped in tooth powder followed by spraying with a pleasant-tasting mouth wash.

If the tongue is coated and the teeth are not clean as usual I inquire into home care. If conditions are very bad I go into the question of diet. It is surprising to see how quickly people, when comfortable, slip into old ruts, and to be successful in prophylaxis one must correct bad habits and get renewed promises of more thorough mastication, etc. Painting the teeth with a disclosing solution and showing where they are neglected, makes the parent more watchful for the coming month. Many dentists think that prophylaxis two or three times a year is sufficient, but the majority of children under my care cannot be left on their own responsibility over two months or they fall back into their old, bad habits.

For many years I have believed that prophylaxis for school children was one of the best ways of counteracting the overwhelming amount of carious teeth, but I am now of the opinion that teaching the children the proper food should also be a part of the work of the dental hygienist.

### HOME ORAL HYGIENE.

Perhaps my teaching of home care is more simple than most of you believe in but I think the results have proven its worth. Upon arising, rinse the mouth and drink a large glass of water. *After breakfast* and *before retiring* brush the teeth thoroughly. Have two brushes and use no *paste, powder nor dental floss.* Always begin with the brush *dry* and go over all the surfaces of the teeth beginning on the gums and brushing toward the cutting edge, that is, down on the uppers and up on the lowers. After going over all the teeth carefully, wet the same brush using plenty of cold water and go over them in the same manner again and again. When finished, wash the brush and hang it up to dry. The object in having two brusshes is to use the same brush only once in twenty-four hours, so that it will be perfectly dry. Children will not brush the back teeth carefully with paste or powder because it gets into the throat and I always find neglected molars where dentifrices are used. The effect of the dry brush is also lost.

If the child is eating enough rough foods and fruit this is sufficient and if he is eating an excess of carbohydrates no paste or powder will save his teeth. There is nothing at present so dangerous to the health of the soft tissues as teaching a child to use dental floss. It is, I believe, the cause of more pyorrhea later in life than the entire neglect of the teeth would be. I am very emphatic in this with the children under my care and you could not wish for harder or more perfect gums, filling the interproximal spaces, and with teeth in such close contact that food never packs between them. If the gums begin to soften, the child is instructed to massage them once a day with camphophenique until they are hard again and to masticate more thoroughly in helping them to be restored to health.

### CONCLUSION

The body might be compared to a wonderful high-powered machine in which all parts must work together. Poor teeth are the broken bearings which slow down the machine and may cause it to be thrown into the junk heap. In studying the conditions surrounding children we must not overlook the need of fresh air, sunlight, exercise and regular habits and lay all the ills to improper feeding. Although as long as the diet is as deficient in vitamines as that of the average family eating white bread, we should expect to find undernourished, anemic, and often rachitic children. To change a child's diet is easy, as illustrated by a five-year-old boy who wanted to stay home and play, so said to his mother, "Why don't you feed me decent, so I won't have to go to the dentist?"

### BIBLIOGRAPHY.

Alfred A. Hess, M.D., *Journal of American Medicine*, March 12, 1921.

George Huston Bell, M.D., *Dental Cosmos*, May, 1921.

Dr. Percy Howe, *Dental Cosmos*, May and August, 1920.

"Newer Aspects of Some Nutritional Disorders," March 12, 1921.

